# Military Methods in Medicine

**Published:** 1915-05-15

**Authors:** 


### The Hospital

The Workers' Newspaper of Administrative Medicine and Institutional Life, Administration, National Insurance and Health.

No. 1508, Vol. LVIII. SATURDAY, MAY 15, 1915. PRICE ONE PENNY

### MILITARY METHODS IN MEDICINE.

It is hardly a matter for surprise to find that the appearance of Sir Almroth Wright in the guise of a colonel has been followed by the eruption of questions which challenge principles that have hitherto been regarded as established and beyond the reach of discussion. Both in professional activities and in social problems he has accustomed us to expect that his arrival on the scene will produce agitations where previously we had enjoyed the calm and peace of settled convictions. If this is true of details and methods it is still more true of the fundamental issues which lie at the heart of things. Proposals which disturb arrangements on the surface are not unwelcome and are easily accommodated under the title of progress. But to shake the very foundations is an act of boldness well calculated to cause alarm even to those who try to keep an open mind. Far be it from us to say that the experience may not in the end be beneficial. The immediate effect, however, is disturbing, and naturally the dovecots are fluttered.

As is a matter of common knowledge, Sir Almroth, in his new position, has been specially concerned with the application of bacteriological methods to the special problem of the treatment of wounds as these are produced on the field of battle. But from his most recent utterance it is evident that he has not restricted his interest to any mere technical inquiry. He has become a part of the complex organisation of the military medical service and, as is his wont, he has surveyed the whole of the machinery and has come to the conclusion that, at least in certain respects, it is capable of improvement. To a generous recognition of many excellences he adds a proposal which he himself describes as a fundamental change in the existing scheme of work. This is no less than the removal from the individual medical officer of a free choice in the treatment to be applied to the patients under his care, and the replacement of this choice by orders and instructions imposed by superior authority. To those of us engaged in civil practice the suggestion has a quality truly revolutionary, and we may feel somewhat timorous lest what begins in military medical practice may come to have a wider application. The thin-end-of-the-wedge argument, however, is never valid when it stands alone, and it is necessary to consider the reasons which are urged in favour of the new proposal.

The reasons advanced are based on the assumption that there are essential differences between civil and military practice, and that the individual freedom which is so suitable to the one is profoundly unsuitable to the other. If this general statement is true, the proposals which grow out of it are of particular importance at the present moment, when numbers of civilian practitioners have suddenly undertaken military duties at the request of the Army authorities. The first difference insisted on is that the problem of projectile wounds and wound infections is an unfamiliar one to the civilian practitioner. Even if this statement is admitted—and a protest against it in so unqualified a shape has already been entered by one of Sir Almroth's colleagues—is it a sufficient reason for replacing the ordinary methods of instruction and argument by the sharp commands of authority? More impressive, perhaps, is the claim that the military medical officer, unlike his civilian brother, has little opportunity of seeing the result of his work. The patients which are under his care to-day—the argument is applied to the position now existing in France—are to-morrow, from the pressure of military necessity, transferred to another hospital as a further stage on their homeward journey. The free exercise of private judgment in such circumstances is not checked by individual personal experience, and action taken in an early stage shows its consequences only to those who have to assume the later responsibility. We venture to think that here is the strong point of Sir Almroth Wright's case. There does seem a reasonable claim for some authoritative guidance for junior men whose immediate duty is mainly to deal with acute emergencies while leaving the essential surgical problem to those who have greater experience and enjoy a less hurried opportunity. If no such guidance exists we agree that it ought to be provided, and in this limited sense the principle advocated carries our convictions.

But Sir Almroth Wright would seem to covet a much wider ambition. He wishes a professional head over the service for treatment, an advisory committee, and a research department, all of which may indeed be most valuable as a means of collating information and spreading knowledge. But if the object is to make every man work, "not as he individually thinks best, but as part and parcel of a great machine," the policy is not without its dangers. Medicine has in its day lived under authority, and a very sterile time it was. There is little temptation to repeat the experience, even though bacteriological laboratories should take the place of the fathers of the profession. Speaking generally, it has been good for practical medicine and good for the public that the freedom of the individual practitioner should be unfettered save by the claim of individual responsibility. To this principle we strongly adhere, though we agree that in the special circumstances of the moment there is room for some modification of it in the limited direction we have above defined. That committees and professional heads should submit reasons and advice all will agree, but commands ought to be kept within a very narrow compass.

